# Supplementary material for: Spatiotemporal Mapping of Online Interest in Cannabis and Popular Psychedelics before and during the COVID-19 Pandemic in Poland
Source: Int J Environ Res Public Health. 2022 May 29;19(11):6619. doi: 10.3390/ijerph19116619 (PMC9180639; doi:10.3390/ijerph19116619)
Supplement: Supplementary file 1 [file ijerph-19-06619-s001.zip › ijerph-1705025-supplementary.pdf]

Table S1. Poland-specific dictionary of the most popular psychedelics.

	Combination of terms
1	2C-B-FLY
2	AL-LAD
3	"ayahuasca"+"*Banisteriopsis caapi*"+"*Psychotria viridis*"+ "*Diplopterys cabrerana*"
4	"DMT"+"N,N-Dimethyltryptamine"+"5-MeO-DMT"+"**dimetylotryptamina**"
5	"DOB"+"2,5-Dimethoxy-4-bromoamphetamine"+"**brolamfetamina**"+"**2,5-Dimetoksy-4-bromoamfetamina**"
6	"dronabinol"+"marinol"+"sativex"
7	"DXM"+"dextromethorphan"+"robitussin"+"**dekstrometorfan**"
8	"ecstasy"+"MDMA"
9	"ergine"+"LSA"+"**ergina**"
10	ibogaine
11	"ketamine"+"ketalar"+"**ketamina**"
12	"LSD"+"lysergic acid diethylamide"+"**dietyloamid kwasu lizergowego**"
13	marihuana+"tetrahydrocannabinol"+"THC"+"**konopie indyjskie**"+" tetrahydrokannabinol"+"Cannabis indica"
14	"mescaline"+"peyote"+"**meskalina**"+"**pejotl**"+"**Jazgrza Williamsa**"+"*Lophophora williamsii*"
15	"methamphetamine"+"crystal meth"+"**metaamfetamina**"
16	Nabilone
17	25*-NBOMe+"25[-x]-NBOMe"+"N-methoxybenzyl"
18	"phencyclidine"+"PCP"+"angel dust"+"**fencyklidyna**"
19	"psilocybin"+"magic mushrooms"+"**psylocybina**"+"*Psilocybe semilanceata*"+"Psilocybe *cubensis*"
20	"Salvinorin A"+"sage of the diviners"+"**szałwia wieszcza**"+"**salwinoryna A**"+"*Salvia divinorum*"

Italic fonts represent species names; bold fonts represent Polish translations of psychedelics.

Table S2. Comparative analysis of the slopes for the time series in Poland.

Psychedelic Substance	Pre-Pandemic Period						Pandemic Pandemic					
	Intercept Coeff.	SE	p-value	Slope Coeff.	SE	p-value	Intercept Coeff.	SE	p-value	Slope Coeff.	SE	p-value
NBOMe	45.029	0.974	<0.001	0.054	0.010	<0.001	58.436	2.218	<0.001	-0.079	0.040	0.052
Cannabis	57.306	1.645	<0.001	-0.030	0.017	0.077	48.376	1.720	<0.001	-0.025	0.031	0.421
DMT	13.373	1.948	<0.001	0.010	0.020	0.626	13.243	1.151	<0.001	-0.019	0.021	0.356
LSD	55.278	2.662	<0.001	-0.007	0.028	0.814	58.559	2.952	<0.001	-0.249	0.053	<0.001
MDMA	26.584	2.038	<0.001	0.128	0.021	<0.001	41.536	2.954	<0.001	-0.088	0.053	0.102
Phencyclidine	29.186	2.542	<0.001	0.019	0.026	0.467	45.858	3.345	<0.001	-0.163	0.061	0.009
Ketamine	18.734	2.790	<0.001	0.056	0.029	0.057	24.297	4.270	<0.001	0.118	0.077	0.130
Mescaline	28.106	2.748	<0.001	-0.071	0.029	0.014	22.636	3.550	<0.001	-0.047	0.064	0.462
Psilocybin	13.966	2.833	<0.001	0.047	0.029	0.112	14.245	4.158	0.001	0.215	0.075	0.005
Ayahuasca	30.209	3.869	<0.001	-0.003	0.040	0.950	32.492	3.829	<0.001	-0.130	0.069	0.064
DOB	46.720	3.811	<0.001	-0.128	0.040	0.001	35.747	19.929	0.076	-0.011	0.092	0.902
DXM	29.973	3.312	<0.001	-0.017	0.034	0.612	-21.616	14.383	0.136	0.213	0.067	0.002
Ergine	28.634	3.160	<0.001	-0.027	0.033	0.417	-13.558	15.302	0.378	0.199	0.071	0.006
Methamphet.	15.550	3.037	<0.001	0.027	0.032	0.385	14.872	3.018	<0.001	-0.006	0.055	0.908
Salvinorin A	13.394	3.043	<0.001	0.024	0.032	0.451	20.044	15.221	0.191	-0.035	0.071	0.621
Dronabinol	21.141	3.303	<0.001	-0.078	0.034	0.024	7.557	12.812	0.557	0.007	0.059	0.902
Ibogaine	6.773	2.383	0.005	0.003	0.025	0.894	9.467	6.953	0.177	-0.033	0.032	0.314
2C-B-FLY	4.604	2.457	0.063	-0.003	0.026	0.898	3.890	3.646	0.289	-0.016	0.017	0.346
Nabilone	0.113	2.029	0.956	0.020	0.021	0.351	-14.349	11.870	0.230	0.079	0.055	0.153
AL-LAD	10.936	3.232	0.001	-0.071	0.034	0.036	-4.099	6.891	0.553	0.023	0.032	0.469

Abbreviations: Coeff: coefficient; SE: standard error.
Pre-Pandemic Period:01-Jan-2017 to 07-Mar-2020; Pandemic Period: 08-Mar-2020 to 01-Jan-2022.

Table S3. Spatial Mapping Correlation with Population Demographics and Interest in the Deep Web: Holistic Period.

Demographics		NBOMe	Cannabis	DMT	LSD	MDMA	Phencyclidine	Ketamine	Mescaline	Psilocybin	Ayahuasca	DOB	DXM	Ergine	Methamphetamine	Ibogaine
Population	r	0.003	0.366	0.407	0.259	**0.506**	0.440	**0.843**	**0.777**	**0.785**	**0.749**	**0.788**	**0.848**	**0.745**	**0.776**	-0.015
	p-value	0.990	0.163	0.118	0.333	0.046	0.088	<0.001	<0.001	<0.001	<0.001	<0.001	<0.001	0.001	<0.001	0.566
GRP	r	-0.384	0.190	0.121	0.229	**0.503**	0.241	**0.610**	**0.674**	**0.823**	**0.770**	**0.821**	**0.771**	0.469	**0.723**	-0.148
	p-value	0.142	0.480	0.655	0.395	0.047	0.369	0.012	0.004	<0.001	<0.001	<0.001	<0.001	0.067	0.002	0.585
HDI	r	-0.044	0.273	0.295	0.253	0.407	**0.445**	**0.779**	**0.718**	**0.892**	**0.819**	**0.891**	**0.890**	**0.676**	**0.710**	-0.017
	p-value	0.872	0.306	0.268	0.344	0.118	0.048	<0.001	0.002	<0.001	<0.001	<0.001	<0.001	0.004	0.002	0.951
Deep Web (i)	r	**-0.543**	0.498	0.416	0.426	0.413	-0.036	0.561	0.447	**0.567**	**0.705**	0.580	**0.611**	**0.460**	0.478	-0.117
	p-value	0.030	0.049	0.109	0.100	0.112	0.894	0.024	0.083	0.022	0.002	0.018	0.012	0.037	0.061	0.666

Significant correlations are in bold fonts.
Due to a lack of data, five substances were excluded (Salvinorin A, Dronabinol, 2C-B-FLY, Nabilone, and AL-LAD).
Abbreviations: Deep Web (i): interest in the deep web; GRP: gross regional product; HDI: human development index; r: correlation coefficient.

Table S4. Spatial Mapping Correlation with Population Demographics and Interest in the Deep Web: Pre-Pandemic.

Demographics		NBOMe	Cannabis	DMT	LSD	MDMA	Phencyclidine	Ketamine	Mescaline	Psilocybin	Ayahuasca	DOB	DXM	Ergine	Methamphetamine	Ibogaine
Population	r	0.166	0.306	**0.559**	0.180	**0.503**	0.236	**0.701**	**0.504**	**0.650**	**0.546**	**0.635**	**0.713**	**0.620**	**0.508**	-0.206
	p-value	0.539	0.249	0.024	0.504	0.047	0.379	0.003	0.046	0.006	0.029	0.008	0.002	0.010	0.045	0.443
GRP	r	-0.244	0.394	0.323	0.332	**0.567**	0.043	**0.595**	0.422	**0.701**	**0.585**	**0.582**	**0.691**	**0.516**	0.476	-0.051
	p-value	0.363	0.131	0.233	0.208	0.022	0.875	0.015	0.103	0.002	0.017	0.018	0.003	0.041	0.062	0.851
HDI	r	0.070	0.282	0.399	0.243	**0.504**	0.137	**0.508**	0.308	**0.623**	**0.544**	0.493	**0.568**	0.415	0.349	-0.168
	p-value	0.796	0.290	0.126	0.384	0.047	0.612	0.044	0.245	0.010	0.030	0.052	0.022	0.119	0.185	0.533
Deep Web (i)	r	**-0.509**	0.439	**0.509**	0.319	**0.654**	0.003	0.329	0.407	0.399	**0.595**	0.214	0.310	0.370	0.446	-0.137
	p-value	0.044	0.089	0.044	0.229	0.006	0.991	0.214	0.118	0.126	0.015	0.417	0.242	0.158	0.083	0.614

Significant correlations are in bold fonts.
Due to a lack of data, five substances were excluded (Salvinorin A, Dronabinol, 2C-B-FLY, Nabilone, and AL-LAD).
Abbreviations: Deep Web (i): interest in the deep web; GRP: gross regional product; HDI: human development index; r: correlation coefficient.

Table S5. Spatial Mapping Correlation with Population Demographics and Interest in the Deep Web: Pandemic.

Demographics		NBOMe	Cannabis	DMT	LSD	MDMA	Phencyclidine	Ketamine	Mescaline	Psilocybin	Ayahuasca	DOB	DXM	Ergine	Methamphetamine	Ibogaine
Population	r	<0.001	0.180	**0.589**	0.290	0.481	0.454	**0.645**	**0.508**	**0.590**	**0.569**	**0.586**	**0.677**	**0.548**	**0.505**	0.482
	p-value	1.000	0.504	0.016	0.276	0.059	0.077	0.007	0.045	0.016	0.021	0.017	0.004	0.028	0.041	0.059
GRP	r	-0.372	0.014	0.363	0.410	0.435	0.089	**0.582**	0.415	**0.691**	**0.592**	0.426	**0.691**	0.339	**0.533**	0.432
	p-value	0.156	0.959	0.167	0.115	0.092	0.742	0.018	0.111	0.003	0.016	0.100	0.003	0.129	0.033	0.095
HDI	r	-0.055	0.234	0.394	0.204	**0.508**	0.401	**0.553**	0.258	**0.549**	**0.571**	0.387	**0.549**	0.369	0.364	0.370
	p-value	0.840	0.348	0.131	0.449	0.044	0.123	0.026	0.335	0.028	0.021	0.139	0.028	0.159	0.166	0.158
Deep Web (i)	r	-0.453	0.333	**0.530**	0.348	0.445	0.010	0.297	0.366	0.489	**0.556**	0.161	**0.523**	0.405	0.396	0.395
	p-value	0.078	0.208	0.053	0.186	0.084	0.972	0.264	0.164	0.055	0.025	0.552	0.038	0.120	0.129	0.130

Significant correlations are in bold fonts.
Due to a lack of data, five substances were excluded (Salvinorin A, Dronabinol, 2C-B-FLY, Nabilone, and AL-LAD).
Abbreviations: Deep Web (i): interest in the deep web; GRP: gross regional product; HDI: human development index; r: correlation coefficient.

Table S6. Binary Indexing of the Spatial Map Correlation for Pre-Pandemic versus Pandemic Period.

Demographics	NBOMe	Cannabis	DMT	LSD	MDMA	Phencyclidine	Ketamine	Mescaline	Psilocybin	Ayahuasca	DOB	DXM	Ergine	Methamphetamine	Ibogaine
Population					-1										
GRP					-1						-1		-1	**+1**	
HDI															
Deep Web (i)	-1				-1							**+1**			

A value of "-1" indicates a change from significant to non-significant; "+1"; indicates a change from non-significant to significant.
